# Raman-Enhanced Spectroscopy (RESpect) Probe for Childhood Non-Hodgkin Lymphoma

**DOI:** 10.28991/scimedj-2020-0201-1

**Published:** 2020-03

**Authors:** Melissa Agsalda-Garcia, Tiffany Shieh, Ryan Souza, Natalie Kamada, Nicholas Loi, Robert Oda, Tayro Acosta-Maeda, So Yung Choi, Eunjung Lim, Anupam Misra, Bruce Shiramizu

**Affiliations:** a Department of Tropical Medicine, Medical Microbiology, and Pharmacology, University of Hawaii, Hawaii, United States.; b Department Molecular Biosciences & Bioengineering, University of Hawaii, Hawaii, United States.; c Hawaii Institute of Geophysics and Planetology, University of Hawaii, Hawaii, United States.; d Biostatistics Core, Department of Complementary and Integrative Medicine, University of Hawaii, Hawaii, United States.

**Keywords:** Lymphoma, Non-Hodgkin Lymphoma, Raman Spectroscopy

## Abstract

Raman-enhanced spectroscopy (RESpect) probe, which enhances Raman spectroscopy technology through a portable fiber-optic device, characterizes tissues and cells by identifying molecular chemical composition showing distinct differences/similarities for potential tumor markers or diagnosis. In a feasibility study with the ultimate objective to translate the technology to the clinic, a panel of pediatric non-Hodgkin lymphoma tissues and non-malignant specimens had RS analyses compared between standard Raman spectroscopy microscope instrument and RESpect probe. Cryopreserved tissues were mounted on front-coated aluminum mirror slides and analyzed by standard Raman spectroscopy and RESpect probe. Principal Component Analysis revealed similarities between non-Hodgkin lymphoma subtypes but not follicular hyperplasia. Standard Raman spectroscopy and RESpect probe fingerprint comparisons demonstrated comparable primary peaks. Raman spectroscopic fingerprints and peaks of pediatric non-Hodgkin lymphoma subtypes and follicular hyperplasia provided novel avenues to pursue diagnostic approaches and identify potential new therapeutic targets. The information could inform new insights into molecular cellular pathogenesis. Translating Raman spectroscopy technology by using the RESpect probe as a potential point-of-care screening instrument has the potential to change the paradigm of screening for cancer as an initial step to determine when a definitive tissue biopsy would be necessary.

## 1. Introduction

Timely and efficient diagnoses of solid tumors require histopathological evaluation of tissue specimens which remains the gold standard of differentiating malignant cells from normal cells. The diagnostic workup of biopsy tissue focuses on morphological and molecular characteristics of individual cells to determine the malignant type which will lead to appropriate therapeutic action. This process has some limitations particularly when timing is important to begin treatment. While new diagnostic technologies such as next generation sequencing have contributed towards improved outcomes and understanding mechanisms of pathogenesis [1], novel tools such as the use of Raman spectroscopy (RS) enhanced by using a portable fiber-optic probe, i.e. Raman-enhanced spectroscopy (RESpect) probe, could be leveraged to provide rapid and real-time assessment of disease [2, 3]. RS utilizes laser-based technology to characterize the biochemical phenotype of tissue without chemical fixatives and markers or stains. Thus, RS could easily pave the way to complement diagnostic paradigms in a timely fashion, particularly in the pediatric setting where access to tissue can be challenging at times [3–6].

Recent published data characterized RS fingerprints on non-Hodgkin lymphoma (NHL) cell lines which provided the foundation for the current report to test the RESpect probe on clinical NHL specimens [7]. While other research groups used RS to analyze adult cancer specimens thus suggesting its role clinically, the goal of the current study was to determine if the RESpect probe could characterize childhood tumors [2, 4, 8, 9]. The rationale for focusing on this population is that the amount of tissue specimen that is often available from infants and children present challenges in diagnosis which in adults can also be challenging [5]. If RS technology can be adapted for real-time diagnostic approaches, it might be possible to employ the RESpect probe clinically without and initial invasive biopsy procedure in situations where timely assessment would be paramount. A potential advantage of the RESpect probe in infants and children to evaluate tumor diagnoses would be to differentiate malignancies non-invasively and leverage the ability to screen in real-time at point of care [4].

RS identifies chemical and molecular fingerprints of materials through inelastic scattering of photons with molecular bond vibrations that results in frequency energy shifts [10–13]. The corresponding vibrational energy is unique to tissue-specific molecular bonds which can characterize intrinsic molecular fingerprints of DNA, protein, and lipid content of specimens. In this manner, RS has the potential to differentiate tumor tissue particularly in a non-destructive approach [2, 4, 10]. To analyze tissue, complex RS instrumentation utilizes a laser source combined with a special microscope to capture scattered light [2, 10]. In order to translate the technology clinically, a RESpect fiber-optic probe was designed so that when the tip of the probe is in contact with tissue, the laser and captured scattered light are sourced at the tip for potential real-time applicability [13]. The RESpect probe has not been used in pediatrics or in childhood cancers.

RS and RESpect probe analyses were undertaken to characterize fingerprints of a spectrum of NHL subtypes and control tissues. The results provided a foundation to validate a real-time application of the portable RESpect probe to assess tissue characteristics in the initial diagnostic work-up and possibly follow-up of tumor response during therapy [12–14].

## 2. Materials and Methods

### 2.1. Tissue Specimens

The study was approved in accordance with the University of Hawaii Institutional Review Board. Snap frozen pediatric NHL and non-malignant tissue specimens were obtained from the Cooperative Human Tissue Network (CHTN), Pediatric Branch, Columbus, OH and stored at −140°C until processed for RESpect analysis, Table 1. Samples were recorded as being obtained between 1992–2007 when listed. CHTN is a prospective procurement entity with a pediatric division which operates under rigorous quality assurance/control standards with institutional pathology reports available to verify diagnosis.

### 2.2. Raman Spectroscopy

Front-coated aluminum mirror sheets (Anomet, Inc., Ontario, Canada) of 25 mm (length) × 25 mm (width) × 0.5 mm (thickness) were cut and cleaned with methanol [15]. Tissue slices were prepared on a sanitized cold block and transferred to the RS facility suspended in chilled 0.9% NaCl solution. Tissues were placed directly on the aluminum substrates for RS analysis. Data were acquired using an RS microscope as previously described [7]. The RS data were captured using a 40-point scan with 15 second exposures and 15 accumulations using a micro-Raman RXN system (KOSI, Inc., Ann Arbor, MI) with a 785 nm laser and automated xyz-microscope stage and 50μm slit width. Each data point was collected with 30mW laser power at the sample. RS instrument function was verified through the measurement of cyclohexane.

For the RESpect probe (EmVision, LLC, Loxahatchee, Florida), sequential acquisition of RS utilized a unique two-component converging lens that overlapped the laser excitation and collection cones. The RESpect probe was interfaced with a modified RS portable system equipped with a 785nm laser (Wasatch Photonics, Durham, NC) controlled using a custom LabView (National Instruments) program and analyzed with Enlighten software [7, 14]. The RESpect probe was focused on the same tissue preparation that was used to capture the standard RS data from the laboratory RS instrument. The RS data were captured with 50mW laser power and an acquisition time of 4500 ms. Probe function was verified through the measurement of acetaminophen at a concentration of 200mg/mL.

### 2.3. Data Analyses

Outlier spectra were removed and each spectrum was manually baseline corrected and normalized in the region of 700 to 1800 cm^−1^ using Grams/AI Spectroscopy Software (Thermo Fisher Scientific, Waltham, MA) [16]. MATLAB was used to convert the spectroscopy files from the RS instrument to excel files. Spectra from the processed data were averaged to produce an RS profile of each sample which produced characteristic unique peaks of the NHL and follicular hyperplasia specimens. Signal to noise ratios were determined in MATLAB using the ratio of the mean over standard deviation of the 1003 cm^−1^ phenylalanine peaks with an average of 6.50 and 7.56 for the RESpect probe and laboratory RS instrument respectively. To compare RS data between different NHL pathologies, Principal Component Analyses (PCA) were conducted for each of the NHL pathologies using the ChemoSpec package in R version 3.5.1. The obtained principal components (PC) were visualized using the first two PCs where the RS peaks were identified as local maxima with signal to noise ratio of 2, span of 40 points each to the left and right to estimate the local variance, and span of 5 points each to the left and right for smoothing.

## 3. Results

RS signature fingerprints were obtained from 11 frozen tissues from 11 different patients. A summary of the range of diagnoses is highlighted in Table 1 which included different childhood NHL subtypes and follicular hyperplasia as non-malignant tissue. There were 3 follicular hyperplasia (FHP), 5 B-cell NHL (B-NHL), and 3 T-cell NHL (T-NHL) cases. The quality assessment data for the CHTN tissue confirmed that the NHL specimens comprised 100% tumor and follicular hyperplasia specimens had 0% tumor.

**Figure 1.**
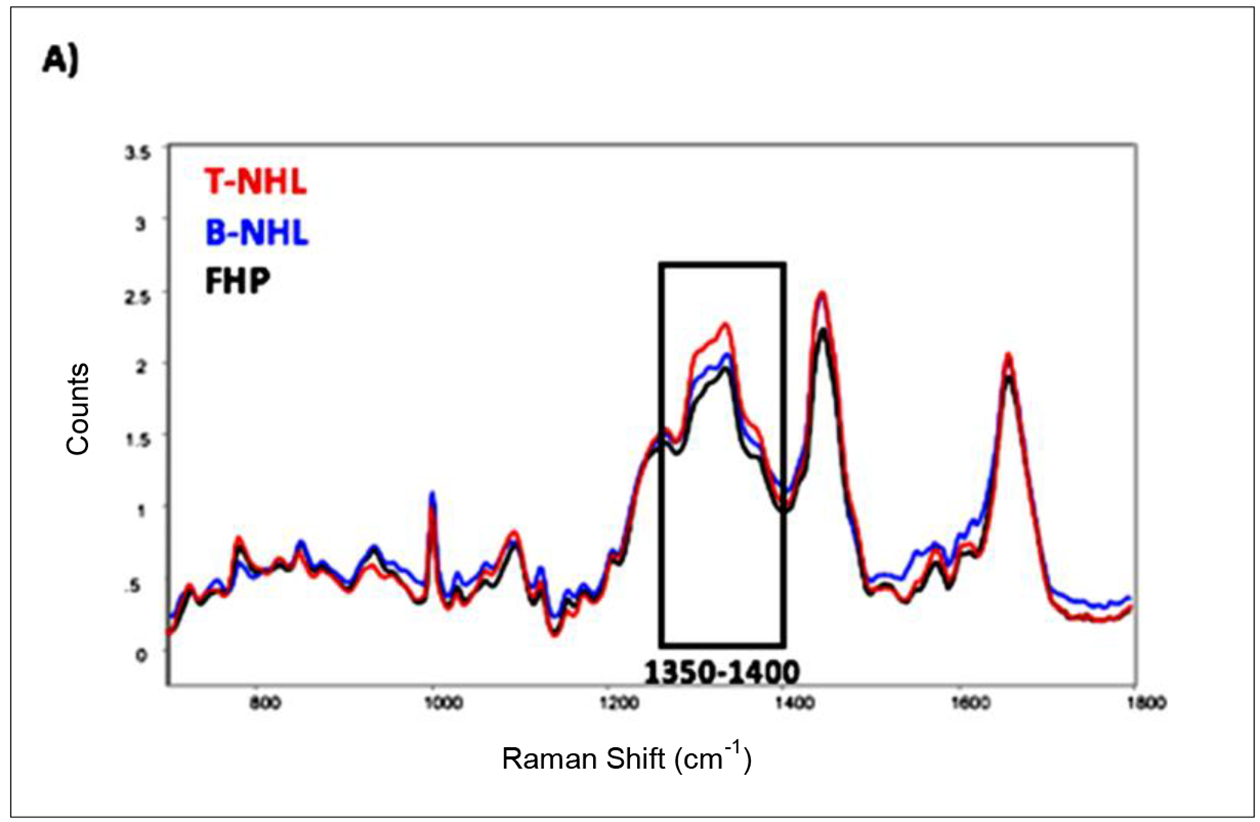

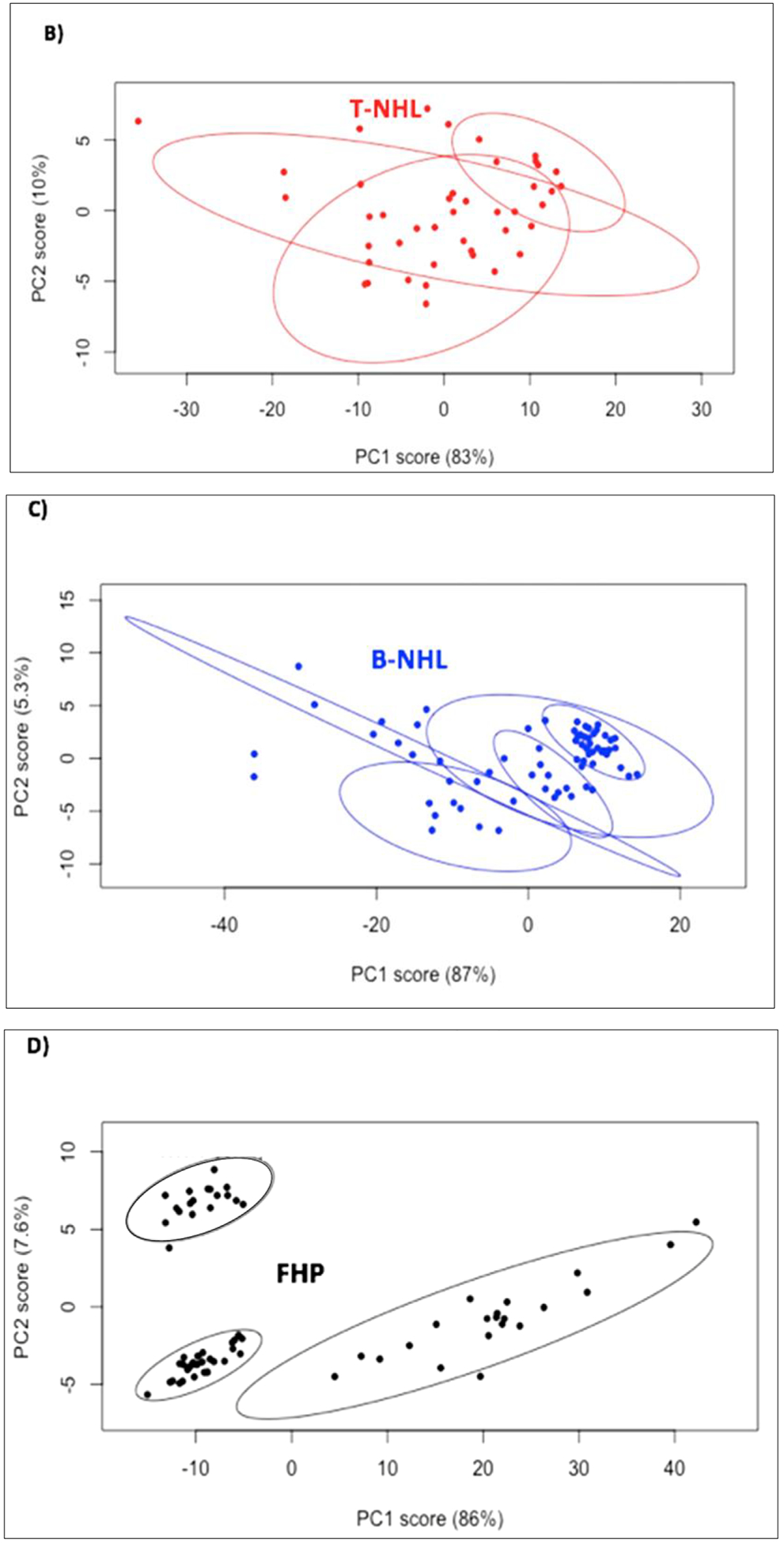
Raman spectroscopy (RS) peaks and Principal Component Analysis (PCA) plots of all NHL specimens categorized into T-cell NHL (T-NHL), B-cell NHL (B-NHL), and follicular hyperplasia (FHP). A) Averaged RS peaks from T-NHL, B- NHL, and FHP highlighting the 1350–1400 cm^−1^ region; B) PCA of NHL subtypes belonging to T-NHL; C) PCA of NHL subtypes belonging to B-NHL; D) PCA of FHP cases.

Analyses were initially completed using the standard RS instrument and the resultant tissue spectra were averaged for all the T-NHL, B-NHL and FHP tissues, Figure 1. Visual comparison of the RESpect signature fingerprints revealed a tryptophan/guanine rich region (1350–1400 cm^−1^) that could potentially be used to differentiate between the three groups as demonstrated in Figure 1A.

### 3.1. Principal Component Analysis (PCA)

RS data from the NHL and FHP tissues were manually baselined followed by PCA for tissues within each classification (T-NHL, B-NHL, and FHP), Figure 1B–D, in which the first two PCs accounting for greater than 92% of variability in the data [17]. These components displayed more related plot clusters between the T-NHL and B-NHL subtypes where all the clusters had overlap in comparison to FHP where no overlap occurred.

### 3.2. RS Instrument and RESpect Probe Peak Comparisons

**Figure 2.**
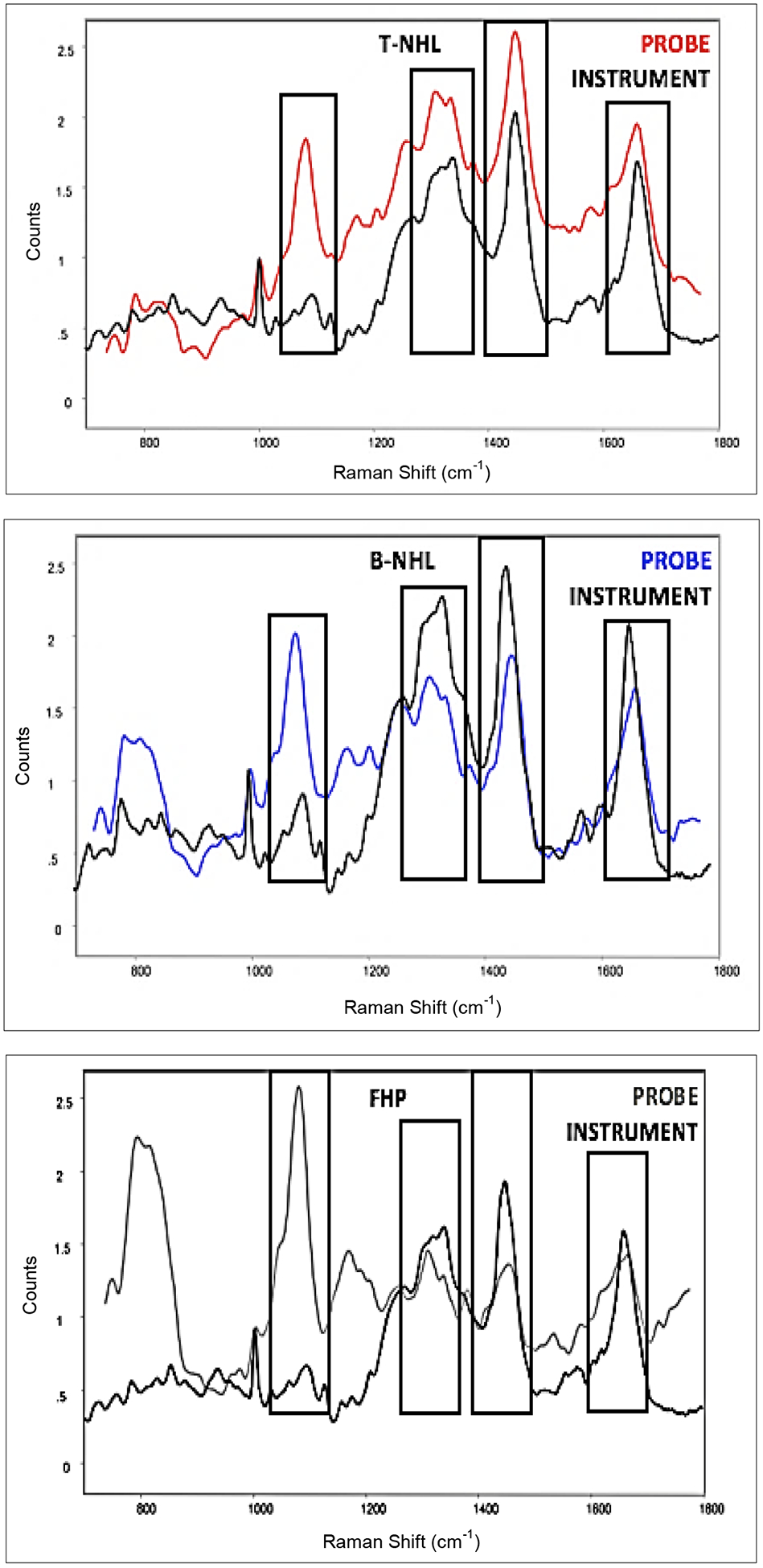
Comparison of averaged Raman spectroscopy (RS) peaks of T-NHL, B-NHL, and FHP from RESpect probe and RS instrument. Primary peaks (1095, 1337, 1448, and 1659 cm^−1^) from the two RS sources are comparable

RS peak data from the standard RS instrument and from the RESpect probe demonstrated comparable primary peaks as shown by the averaged RS instrument and RESpect probe spectra for each group, Figure 2. Focusing on primary peaks with the highest intensity (1095, 1337, 1448, and 1659 cm^−1^) which are comparably measurable by the standard RS instrument and RESpect probe demonstrated the potential of using the RESpect probe for possible future studies.

## 4. Discussion and Conclusion

This study reports for the first time the use of RS technology to discriminate childhood NHL subtypes by standard RS instrumentation and by RESpect probe. While RS technology has previously characterized fingerprints of malignant tissues, characterizing RS fingerprints of childhood NHL subtypes has not been previously reported nor has the RESpect probe been tested on these types of cancers [2, 8, 18]. RS scanning analyzes relatively large specimen volumes to average the information from large numbers of cells [19, 20]. The RS fingerprints contain spectral bands representing molecular modes of vibration of molecules within the tissue. Information from the unique RS fingerprints of pediatric NHL tissues compared to follicular hyperplasia tissue has the potential to contribute information on molecular cellular pathogenesis and mechanisms of malignant transformation which could be applied to discover novel treatment strategies [2, 4, 11].

The spectra of the hyperplastic tissue cells shared similar peaks to NHL tissue which could be assigned to cellular constituents (DNA/RNA, proteins, lipids, carbohydrates) with varying intensities. However, the RS PCA plots of the T-NHL and B-NHL subtypes showed more similarity between one another compared to the non-malignant follicular hyperplasia PCA plots that are more heterogeneous in nature. While similarity occurred between plot clusters within T-NHL and B-NHL, there were non-overlapping areas that revealed some degree of dissimilarity between patients and NHL subtypes. The use of PCA in the analytical algorithm has the potential to provide a more clinical translational approach to deciphering the raw data [4, 16, 21].

This study demonstrated that RS could identify a spectrum of pediatric NHL and follicular hyperplasia. The focus of the current study was to define pediatric NHL RS fingerprints which could be used as markers of disease and to test the feasibility of using the RESpect probe in this setting. A limitation of the study was that the pediatric NHL specimens were comprised of 100% tumor therefore the sensitivity of RS for tissue with less than 100% tumor involvement will need to be assessed in the future. An additional limitation was the small number of cases representing the different types of childhood NHL.

Previous published data demonstrated the feasibility of identifying unique RS fingerprints of pure populations of malignant cells compared to normal cells from in-vitro cell cultures [7]. The current study expands the technology showing unique RS fingerprints across the spectrum of pediatric NHL subtypes. The data have implications for future diagnostic use and prognosis as well as identifying new therapeutic targets for B-NHL. Integrating RS diagnostic fingerprinting in routine cancer diagnostic paradigms could be an innovative future approach to enhance the translation of RS towards new diagnostic prospects [11]. Because RS relies on Raman scattering of radiation fractions by molecules from an incident beam based on chemical structures of molecules, the technology has the capability of being applied to tumor tissue to provide insight at the molecular level [21]. A novel application of RS deserving assessment in the clinical setting is applying a portable RESpect probe that could be used in the clinic setting for initial rapid assessment of tissue for differential diagnosis [12, 13]. Similar RESpect probes have been tested in clinical research during endoscopy and other adult clinical settings [12, 13]. An advantage of a portable RESpect probe in the clinical setting could be its potential front-line application and point-of-care application to assess infants, children and adolescents presenting with potential malignancies.

## Figures and Tables

**Table1.** Tissue characteristics

Tissue	Age	Sex	Tumor Site	Pathology	Tumor Vs Necrosis %	Tumor Vs Stroma %
1	13	F	Lymph Node	Follicular hyperplasia	0	0
2	11	F	Lymph Node	Follicular hyperplasia	0	0
3	6	M	Lymph Node	Follicular hyperplasia	0	0
4	3	M	Abdomen	Burkitt	100	100
5	19	M	Retroperitoneum	Burkitt-like	100	70
6	5	M	Ileum	Burkitt-like	100	90
7	20	M	Retroperitoneum	Recurrent B-cell	100	100
8	19	M	Lymph Node	Diffuse large B-cell	100	100
9	18	F	Lymph Node	Diffuse, large T-cell	100	100
10	18	M	Lymph Node	T-cell lymphoblastic	100	80
11	15	M	Lymph node-left	T-cell lymphoblastic	100	100
